# Automated analysis of lipid drug-response markers by combined fast and high-resolution whole cell MALDI mass spectrometry biotyping

**DOI:** 10.1038/s41598-018-29677-z

**Published:** 2018-07-26

**Authors:** David Weigt, Denis A. Sammour, Timon Ulrich, Bogdan Munteanu, Carsten Hopf

**Affiliations:** 1 Center for biomedical Mass Spectrometry and Optical Spectroscopy (CeMOS), Mannheim University of Applied Sciences, Paul-Wittsack Str. 10, 68163 Mannheim, Germany; 2 HBIGS International Graduate School of Molecular and Cellular Biology, Heidelberg University, Im Neuenheimer Feld 501, 69120 Heidelberg, Germany

## Abstract

Recent advances in matrix-assisted laser desorption/ionization (MALDI) mass spectrometry have enabled whole cell-MALDI mass spectrometry biotyping of drug-treated cultured cells for rapid monitoring of known abundant pharmacodynamic protein markers such as polyacetylated histones. In contrast, generic and automated analytical workflows for discovery of such pharmacodynamic markers, in particular lipid markers, and their use in cellular tests of drug-like compounds are still lacking. Here, we introduce such a workflow and demonstrate its utility for cellular drug-response monitoring of BCR-ABL tyrosine kinase inhibitors in K562 leukemia cells: First, low-molecular mass features indicating drug responses are computationally extracted from groups of MALDI-TOF mass spectra. Then, the lipids/metabolites corresponding to these features are identified by MALDI-Fourier transformation mass spectrometry. To demonstrate utility of the method, we identify the potassium adduct of phosphatidylcholine PC(36:1) as well as heme B, a marker for erythroid differentiation, as markers for a label-free MALDI MS-based test of cellular responses to BCR-ABL inhibitors. Taken together, these results suggest that MALDI-TOF mass spectrometry of lipids and other low molecular mass metabolites could support cell-based drug profiling.

## Introduction

Since the first description of species-specific small-molecule mass spectrometry (MS) fingerprints obtained by whole cell measurements of bacteria in 1975^1^, matrix-assisted laser desorption/ionization (MALDI) MS fingerprinting (also referred to as biotyping) has been established as an important tool in environmental microbiology and for clinical identification of pathogenic bacteria^2–4^. Recently, MALDI-TOF MS fingerprinting methods have been transferred to whole mammalian cells suggesting that they may – in analogy with MALDI-TOF MS-based biochemical assays – eventually be useful as cell assays in pharmacology^5,6^. Drug profiling based on whole cell- (WC-) MALDI-TOF MS protein biotyping workflows using either known abundant protein markers such as polyacetylated histone H4 or defined sets of *m/z* values corresponding to uncharacterized proteins have been established^7,8^. In contrast, generic workflows for development of label-free WC-MALDI-TOF MS cellular tests that utilize small molecule lipid/metabolite markers for determination of IC_50_ values have not been described yet. Therefore, MALDI MS as a fast, sensitive and rather impurity-tolerant method^9^ warrants consideration as a valuable tool for lipid-based whole cell assessment of drug-responses.

Whereas applications of MALDI-TOF MS-based lipid/metabolite fingerprinting of mammalian cell extracts have been described in several fields including bioprocess engineering^10^, to date few studies describe MALDI-TOF MS lipid/metabolite fingerprinting of non-extracted mammalian cells. Notable examples include cardiolipin fingerprinting of leukocytes as a screening tool for Barth syndrome^11^, and lipid profiling for characterization of breast cancer cell lines or of human skin fibroblasts with Parkin mutations in Parkinson’s disease research^12,13^. In a single notable pharmacology-related study, both WC-MALDI MS protein and lipid fingerprints of benign prostatic hyperplasia as well as prostate cancer cells treated with a single concentration of the peptide anti-cancer drug Degarelix were compared^14^. However, WC-MALDI-TOF MS has not been applied to quantitatively monitor concentration-responses of small molecule drugs and to determine cellular drug potencies.

Here, we present a new computational WC-MALDI-TOF MS workflow that automatically extracts low mass *m/z* features that indicate lipid/metabolite concentration-responses in cultured cells. The corresponding lipids/metabolites are then elucidated by MALDI-Fourier transform ion cyclotron resonance (FTICR) MS. As an example, this workflow was applied for determination of IC_50_ values for the anti-leukemia drug imatinib and other BCR-Abl tyrosine kinase inhibitors in the chronic myelogenous leukemia (CML) cell line K562. Evidence for imatinib-induced lipidome changes had been presented in a triomics study of BCR-ABL-positive H929 multiple myeloma cells^15^. In this study, we identified the potassium adduct of the phosphatidyl choline PC(36:1), and heme B, a marker for erythroid redifferentiation of CML cells, as markers of BCR-ABL potency that could be used for IC_50_ determination.

## Results

### A computer-aided workflow for development of cellular tests of drug concentration-response

To date, few WC-MALDI-TOF MS biotyping-based workflows with potential utility as drug profiling tests have been established, most of which are based on the analysis of protein fingerprints^7,8^. While MALDI MS imaging of low molecular mass metabolites and lipids is a wide-spread tool in chemical biology and drug discovery^16,17^. WC-MALDI-TOF MS biotyping methods that measure lipids or other low molecular mass metabolites as a means for cell-based determination of full drug concentration-response curves have not been described yet.

**Figure 1:**
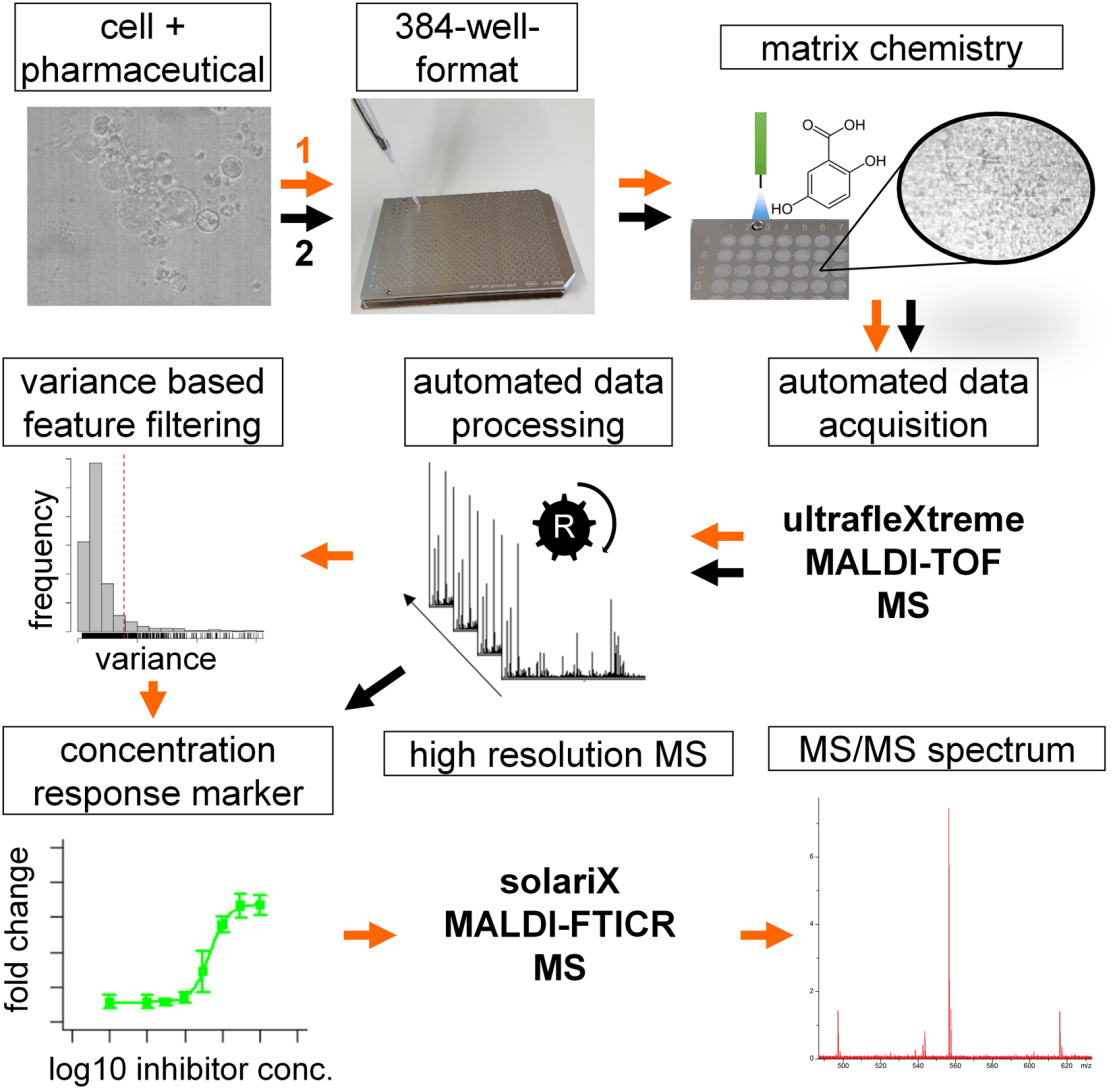
Schematic illustration of the workflows for identification of candidate lipid drug-response markers by MALDI-FTICR MS and their fast measurement by whole cell MALDI-TOF MS biotyping. In concentration-response studies, cells are treated with defined concentrations of an inhibitor and spotted onto a MALDI target plate in 384-well format. The pre-spotted target plate is coated with DHB-matrix using a SunCollect sprayer. MS-spectra are acquired in automated fashion using an ultrafleXtreme MALDI-TOF/TOF MS instrument. Spectra are imported into the R programming environment for automated data preprocessing and evaluation. Fast feature extraction of putative concentration-dependent *m/z* is performed using *m/z* feature-wise variance analysis. Based on sigmoidal curve fits, extracted *m/z* features are evaluated for suitability as concentration-response markers. Structural analysis of candidate marker lipids is performed by ultra-high resolution tandem mass spectrometry using a 7 T Solarix XR FTICR (orange workflow). The identified marker can be used to evaluate drug-potencies of novel pharmaceuticals (black workflow).

To this end, we developed a generic workflow for cell-based MALDI-TOF MS compound profiling (Fig. 1). For systematic method development guided by design of experiment principles, we used scores such as ${J}_{{overlap}}$, which reflects both the scattering of hyperspectral data in PCA space as well as the efficiency of discrimination between different data sets^18^ (see Suppl. Methods for more detail). For comparison of performance of preparation protocols, the ability to separate three cancer cell lines, the leukemia lines K562 and HL60 and the gastrointestinal stromal tumor (GIST) cell line T1, in PCA space was tested. Comparison of *J*_*overlap*_ scores was employed several times to select the most appropriate experimental setup (Suppl. Figs S1–S4).

Moreover, previous work described adjustment of cell numbers and, thus, analyte-to-matrix ion intensity ratio as a key factor to reproducibly work with the same amount of biological material^19,20^. Therefore, we sought to optimize the number of cells spotted per measuring spot in the final step of workflow development. For decision-making and to quantitatively assess the desired suppression of matrix ion signals by sufficiently high lipid content per MALDI target spot, we employed a modification of the previously described score for the matrix suppression effect (MSE) by lipids^21,22^, ${{MSE}}_{{mod}}$. We performed a cell dilution series to optimize the matrix-to-analyte ion intensity ratio (Suppl. Fig. S5**)**. As evident in gel view of the MS spectra of the cell dilution study, increasing cell numbers per measuring spot effectively suppressed matrix ions (Suppl. Fig. S5a). Accordingly, the ${{MSE}}_{{mod}}$ score increased and reached a plateau at about 5,000 cells per measuring spot (Suppl. Fig. S5b). Since the number of detected analyte signals did not increase above 5,000 cells per spot, we decided to use this cell number throughout this study. Scatter plot analysis of all peaks of two independently prepared K562 cell aliquots revealed a good coefficient of determination (R^2^ = 0.92, Suppl. Fig. S6) suggesting acceptable repeatability of the MS fingerprints.

### Automated MALDI MS data acquisition and identification of markers of cellular responses to BCR-Abl tyrosine kinase inhibitors

Earlier work from this laboratory has established the use of histone acetylation-specific mass shifts in whole cell protein MS fingerprints as response markers for histone deacetylase inhibitors^7^. These fingerprints are robust enough to determine cell-based concentration-response curves and pIC_50_ values in a label-free manner that are consistent with reported values obtained in conventional assays. Similar concentration-response studies for small molecules are still lacking.

**Figure 2:**
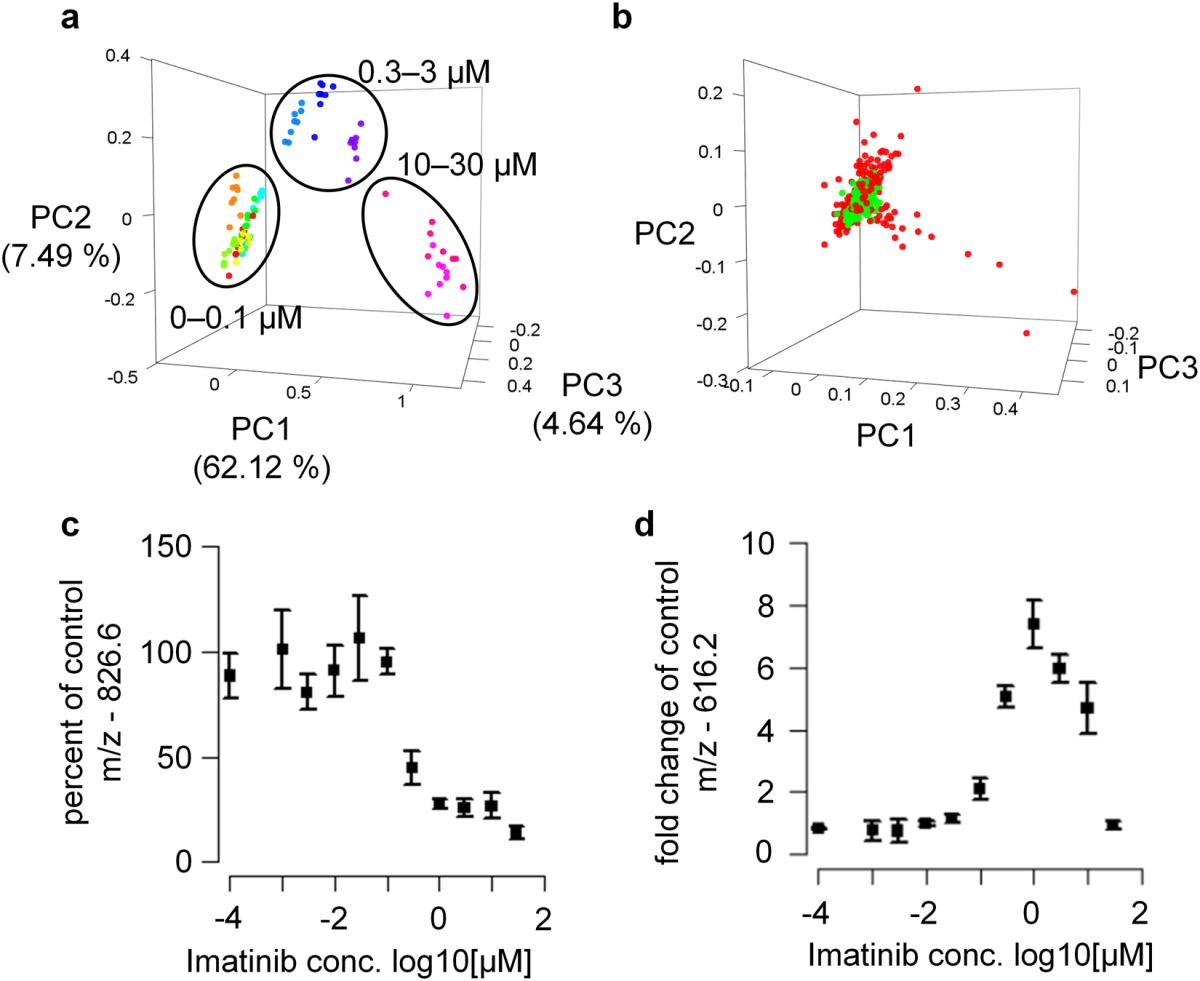
Small molecule fingerprinting enables monitoring of Imatinib concentration- response in K562 cells. Mass spectra of K562 cells treated with various concentrations of Imatinib were acquired on an ultrafleXtreme. (**a**) R language was used for feature extraction (S/N >5, *m/z* 200–2,000) and PCA calculation. Three groups of samples could be distinguished corresponding to low, intermediate and high Imatinib concentrations. Technical replicates of cells treated with the same concentration are illustrated in the same color. Groups corresponding to different concentrations are colored by a rainbow color scheme starting from the lowest concentration in red to the highest concentration in violet. (**b**) PCA loadings plot of A shown to illustrate the ability of variance-based feature filtering to automatically detect high-varying features impacted by drug administration (red). (**c**,**d**) Concentration-responses of two extracted features were plotted using R (mean ± standard deviation for N = 3 biological replicates prepared in different weeks).

To this end, we aimed to identify lipids suitable for cellular tests. We studied the effect of BCR-Abl tyrosine kinase inhibitors on MS lipid/metabolite fingerprints in K562 cells. Cells were treated with various concentrations of imatinib and measured according to the developed workflow. Initial experiments showed that prolonged drug treatment increased the differences in the imatinib-treated cells (Suppl. Fig. S7). After 72 h of treatment, there was evidence of widespread cell death. Therefore, concentration-response experiments were performed over a 48 h incubation period. Data of imatinib-treated cells was analyzed in PCA space, where it formed three groups corresponding to low, intermediate and high imatinib concentrations (Fig. 2a). We were interested in isolating features that are most appropriate to monitor concentration-responses to BCR-Abl inhibitors. Our data processing pipeline was built to extract both increasing, decreasing and biphasic concentration responses. To omit false positive features, low variance features were excluded. This enabled a fast overview on the data and also reduced computation time.

The automated variance-based feature filtering was able to distinguish low- and high-varying relevant *m/z* features (illustrated in green and red in the PCA loadings plot of Fig. 2b, respectively) excluding the former and retaining the latter for subsequent concentration-response fitting analysis. Among the features that showed a decreasing concentration-response, the response curve fit of a feature at *m/z* 826.6 showed the highest coefficient of determination (R^2^ = 0.95, Fig. 2c). Except for the compound itself, none of the features displayed a monotone ascending curve throughout the entire concentration range (i.e. up to 30 µM imatinib). *m/z* 616.2 showed a biphasic concentration-response that likely corresponds to toxicity at high drug concentrations (Fig. 2d). These drug responses were reproducible in three replicate experiments performed weeks apart. We therefore decided to further characterize these two potential response markers.

For any potential protein WC-MALDI-TOF MS fingerprinting-based response marker, which is typically identified in low resolution linear positive mode mass spectra, protein identification would be rather difficult, since such a peak may not represent a full length protein but an unknown fragment which makes correlations with bottom-up proteomics workflows difficult. In contrast, FTICR MS provides a means for direct identification of low molecular mass compounds. The candidate marker molecules were therefore remeasured and fragmented using a SolariX FTICR with resolving power of 1,000,000 at *m/z* 200. Spots that exposed highest feature of interest intensity in TOF-data were chosen for re-analysis. Ultra-high resolution remeasurement of the feature TOF-*m/z* 616.2 revealed a more precise monoisotopic *m/z* of 616.1767 and an M + 1 isotope at *m/z* 617.1800 (Suppl. Fig. S8a).

**Figure 3:**
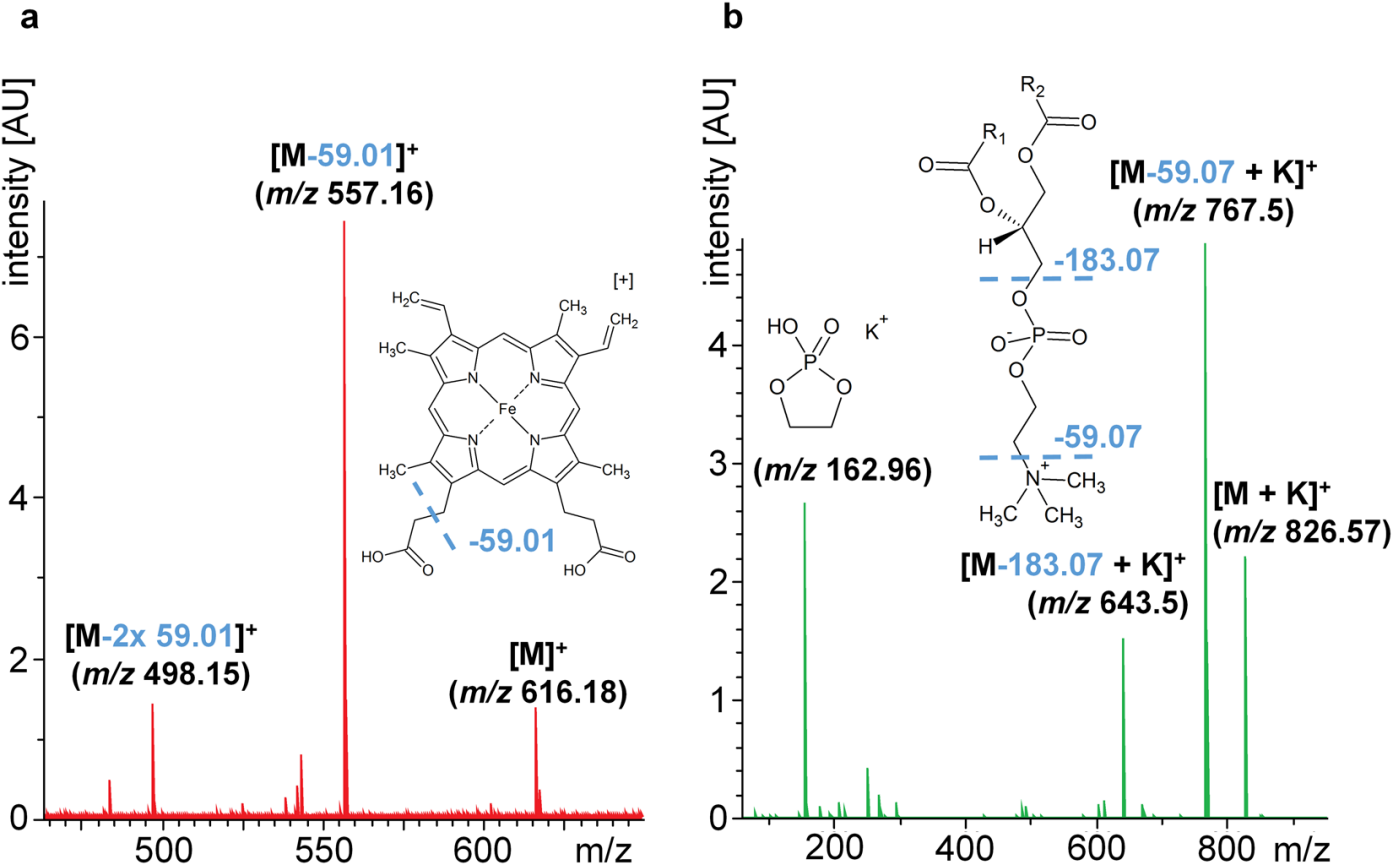
Identification of candidate response markers using ultra-high resolution FT-ICR MS/MS. Spots that show highest feature of interest intensity were re-measured and fragmented using a SolariX 7 T XR FTICR mass spectrometer with R = 500,000. For this reason a spot containing cells treated with 1 µM Imatinib was used for re-measurement and fragmentation of the feature *m/z* 616.1766 (**a**) and a spot containing DMSO-treated cells was used for fragmentation of the feature *m/z* 826.5722 (**b**).

The top hit in a human metabolome database (www.hmdb.ca) search was heme B with a mass accuracy of 1 ppm. Other search results were not considered, because their proposed mass accuracy was above 3 ppm. Note that the hmdb lists the neutral molecular mass of heme (*m/z* 616.1773). Subtraction of the mass of one electron to yield the single positively charged molecule results in *m/z* 616.1767. Fragmentation confirmed that the feature at TOF-*m/z* 616.2 indeed is heme B (Fig. 3a). Fragment masses corresponding to the heme B molecule upon neutral losses of two CH_2_COOH carboxylic acid side chains were found. Signals in the fragmentation spectra were assigned with a mass accuracy of ~1 mDa. Measured isotopic distribution corresponded well with the predicted isotopic distribution of the identified molecule (Suppl. Table S1a).

Ultra-high resolution remeasurement of the second selected TOF-feature *m/z* 826.6 revealed a more precise monoisotopic *m/z* of 826.5722 as well as M + 1 and M + 2 at *m/z* 827.5755 and 828.5789, respectively (Suppl. Fig. S8b). The only acceptable hmdb search result with a mass accuracy below 3 ppm was the potassium adduct of PC(36:1). Search results with a proposed mass accuracy above 3 ppm were not considered. Also search results that proposed uneven numbers of carbon atoms in lipid side chains were excluded. We confirmed that the TOF-feature at *m/z* 826.6 is a phosphatidylcholine by fragmentation (Fig. 3b). We found masses corresponding to neutral loss of trimethylamine N(CH_3_)_3_ and fragments of the polar head group C_2_H_5_PO_4_^23^. Measured isotopic distribution corresponds well with the predicted isotopic distribution of the identified molecule (Suppl. Table S1b).

### Heme B- and PC(36:1) + K^+^-based cellular tests of BCR-Abl potency

**Figure 4:**
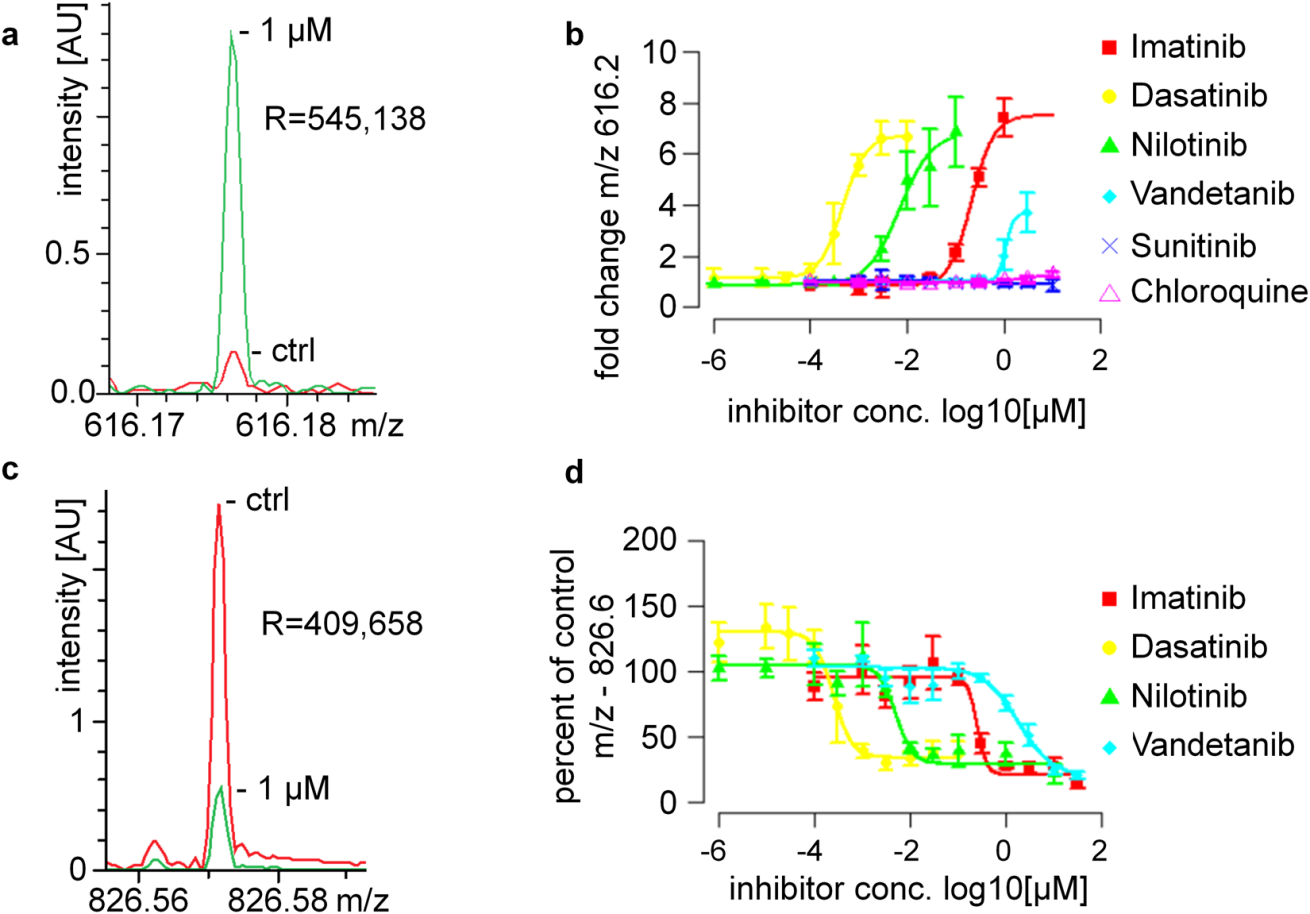
Small molecule MALDI MS fingerprinting enables quantitative comparison of drug responses in K562 cells. (**a**,**c**) FT-ICR spectra of Heme B (m/z_meas._: 616.1767, Δm: 0.3 ppm) (**a**) and PC(36:1) K^+^ (m/z_meas._: 826.5722, Δm: 0.1 ppm) (**c**) signals in vehicle control (red) and Imatinib-treated (green) K562 cells. (**b**,**d**) Concentration-response curves derived from whole cell MS fingerprints recorded by MALDI-TOF MS enable comparison of EC_50_ values for different tyrosine kinase inhibitors and control substances. (**b**) Heme B induction enables monitoring of erythropoiesis in K562 cells. R was used to plot *m/z* 616.2 feature intensity. (mean ± standard deviation for N = 3 biological replicates prepared on different days). (**d**) Monitoring of PC(36:1) + K^+^ enables comparison of different BCR-Abl tyrosine kinase inhibitor potencies. R was used to plot *m/z* 826.6 feature intensity for different BCR-Abl tyrosine kinase inhibitors (mean ± standard deviation for N = 3 biological replicates prepared on different days).

Concentration-responses of the features at *m/z* 616.2 and 826.6 was not only observed in MALDI TOF MS data but also in MALDI FTICR MS data (Fig. 4a,c). Moreover, both marker molecules showed concentration-responses for additional tested inhibitors including the BCR-Abl inhibitors dasatinib, nilotinib and, weakly, vandetanib, but not for the unrelated anti-malaria drug chloroquine or the non-selective but not BCR-Abl targeting tyrosine kinase inhibitor sunitinib (Fig. 4b,d). Concentration-dependent decrease of the feature *m/z* 826.6 enabled the comparison of drug potencies of different tyrosine kinase inhibitors (Fig. 4d) and the deduction of drug pIC_50_ values (Suppl. Table S2). The pIC_50_ ranged from 5.2 for sunitinib to 9.6 for dasatinib. As expected, no inhibition by chloroquine was observed. Overlay of low and high resolution mass spectra confirmed that in the mass range covered by the low resolution signals of heme B and PC(36:1) + K^+^ only one according high resolution mass feature was present (Suppl. Fig. S9). Interfering signals for these specific examples were not observed.

The acquired MALDI-TOF spectra of whole cells contain multiple signals (Suppl. Fig. S10). The two presented features, extracted by our data analysis pipeline seem to be of general validity to evaluate the potency of tyrosine kinase inhibitors in K562 cells. Heme B induction is a known marker for erythropoiesis, i.e. differentiation, in K562 cells^24^ and a key measure of functional drug response of these cells to BCR-Abl inhibitors. Whether there is a similar BCR-Abl-selective functional link between engagement of the BCR-Abl drug target and the observed decrease in PC(36:1) + K^+^ is presently unknown. Quantitative changes in the potassium adducts of PC(34:1) and PC(36:1) have been observed in many studies including in MS imaging. However, with a view to technical applications in cell culture, parallel measurement of heme B and PC(36:1) + K^+^ enables both monitoring of erythroid differentiation as a defined BCR-Abl dependent response and comparison of different drug potencies, respectively.

## Discussion

Here we present the development of an innovative workflow combining both reproducible sample preparation and a standardized data analysis pipeline. We were able to automatically acquire and evaluate drug concentration-responses of lipids/metabolites in whole cells, which to our knowledge has not been performed before. Finally, the switch from a fast data acquisition to a high resolution remeasurement enabled the identification of candidate response markers.

The proposed workflow complements recent advances in MALDI MS-based compound screening in biochemical assays up to 1536- and 6144-assay formats^25–27^. This very rapid technological paradigm shift has been fostered by availability of very fast MALDI-TOF mass spectrometers as well as supporting automation. Nevertheless, it has been argued that such an approach would be difficult to adapt for cell based screening applications^28^. Among other things, ion suppression effects may be difficult to deal with in a routine application setting, and the dynamic range of the assays might be too limited. In this study, we indeed observed a limited dynamic range (3–4-fold) for the decrease in *m/z* 826.6, but a more pronounced one (>6-fold) for *m/z* 616.2 suggesting general feasibility. In the present study, WC-MALDI-TOF MS concentration-responses enable the monitoring of several markers at once such as functional markers of K562 cell differentiation and technologically useful markers for potency evaluation of different inhibitors.

Our understanding why PC(36:1) + K^+^ may be well suited to monitor concentration-responses of tyrosine kinase inhibitors is presently limited and requires follow-up studies. We hypothesize that decrease of this marker may be cell death-related. On the one hand, cell death-related pathways induce lipid-degrading enzymes^29^. An overall reduction of mature lipids would, therefore, be expected. Nevertheless, some phospholipids show a rather stringent signal decrease, while other lipids do not show any concentration-response. It has been known for some time that apoptotic cells loose intracellular potassium in huge amounts, which is accompanied with a dysfunction of the Na/K-ATPase^30^. Therefore, apoptotic potassium loss might cause a decrease of K^+^ adduct formation in the MALDI ionization process.

The current study presents the two features with the most striking concentration-responses. Other concentration-dependent features were observed. In general, the number of lipid/metabolite features that are assessable with this method depends on the repertoire expressed in a given cell type as well as several other parameters like pH, solvent- and MALDI matrix composition that would have to be optimized during assay development. In principle, other molecule classes such as lysophospholipids, sphingomyelin or negatively charged ions can be measured. Therefore, MALDI MS cell-based assays can utilize a broad range of potential target molecules for monitoring concentration-responses.

## Materials and Methods

### Chemicals

All reagents were of HLPC grade. Acetonitrile (ACN) and trifluoroacetic acid (TFA) were purchased from Merck (Darmstadt, Germany). Dimethylsulfoxide (DMSO; cell culture grade) was obtained from Sigma Aldrich (Munich, Germany). Milli-Q water (ddH_2_O; Millipore) was prepared in-house. 2,5-Dihydroxybenzoic acid (DHB), 6-Aza-2-thiothymine (ATT) and the peptide MALDI-MS calibration standard mix (cat. no. # 8222570) were purchased from Bruker Daltonics (Bremen, Germany). Imatinib Free Base (Cat. No. I-5577), Dasatinib Free Base (Cat. No. D-3307), Nilotinib Free Base (Cat. No. N-8207), Vandetanib (Cat. No. V-9402), Sorafenib Free Base (Cat. No. S-8599) and Sunitinib Malate Salt (S-8803) were purchased from LC Laboratories (Woburn, USA); Chloroquine diphosphate salt (Cat. No. C6628) was obtained from Sigma-Aldrich. Olanzapine (Cat. No. S2493) and Romidepsin (Cat. No. S3020) were obtained from SelleckChem.

### Cell culture and inhibitor treatment

K562 cells (Cell Lines Service GmbH, Eppelheim, Germany) were cultivated in RPMI-1640 medium supplemented with 10% FCS (v v^−1^), 2.5 g L^−1^ glucose, 10 mM HEPES, 2 mM L-glutamine, 1 mM sodium pyruvate, and 1 × penicillin/streptomycin. Prior to drug treatment, K562 cells were seeded at 0.25*10^6^ cells mL^−1^ in 24-well plates. On the following day, cells were treated with compound. After 48 h, cells were transferred to Eppendorf cups and spun down at 2,000 rpm on a benchtop centrifuge for 5 min at 4 °C. Culture supernatants removed completely. Dry cell pellets were snap-frozen in liquid nitrogen and stored at −80 °C.

### MALDI-TOF and MALDI-FTICR mass spectrometry sample preparation and data acquisition

Cell aliquots were resuspended at 5,000 cells µL^−1^ in 50% (v v^−1^) ACN in ddH_2_O. One µL of each suspension was applied to a 384-well ground steel target plate (Bruker Daltonics), and eight technical replicates were prepared. DHB-matrix was resuspended at 20 mg mL^−1^ in 50% (v v^−1^) ACN in ddH_2_O supplemented with 0.5% TFA. Dried sample spots were spray-coated with DHB-matrix using a SunCollect sprayer (SunChrom, Friedrichsdorf, Germany). The spray protocol included a spray-head velocity set to 900 mm min^−1^ at a height of 2.8 cm. The distance between sprayed lines was 2 mm. The matrix flow rate was set to 30 µL min^−1^.

The target was then measured by an ultrafleXtreme MALDI-TOF mass spectrometer equipped with a 2 kHz Smartbeam II laser (Bruker Daltonics). Measurements were performed in reflector positive ion mode in a mass range of *m/z* 200–2,000. The sum of 4,000 laser shots per measuring spot was acquired at 40 different positions in random walk mode. The laser focus was set to “medium” on the ultrafleXtreme. The sampling rate was 5 giga samples s^−1^. Data acquisition was controlled by the AutoXecute function of the flexControl 3.4 software (Bruker Daltonics). External cubic calibration was performed using the peptide calibration standard II (Bruker Daltonics) spiked with the small molecular mass compounds Olanzapine ([M + H^+^]^+^ *m/z* 311.1325), Sunitinib ([M + H^+^]^+^ *m/z* 397.2034), Romidepsin ([M + H^+^]^+^ *m/z* 540.2021) and Sorafenib ([M + H^+^]^+^ *m/z* 463.0779). The external calibration spot was in close proximity to the sample spots. To evaluate intra assay-reproducibility, eight technical replicates of each sample were applied per target plate, and all spots were measured on the same day. Preparation and measurement of the same sample on different days for evaluation of inter-assay reproducibility is also referred to as technical replicates, but with an indication of the time that passed between the measurements. Samples that originate from different passage numbers of cultured cells are referred to as biological replicates.

Ultra-high resolution measurements were performed using a solariX 7T XR FTICR (Bruker Daltonics). Data were recorded in positive ion mode using ftmsControl 2.1.0. Laser intensity and shot number per spot were adjusted to obtain signal intensities in the range of 10^8^–10^9^ AU. Ions were detected in the *m/z* range 150–5,000, using a 4 M transient in AMP mode. For ion transfer, voltages of funnel 1 and skimmer 1 were adjusted to 150 V and 15 V, respectively. The funnel RF amplitude was adjusted to 150 Vpp. The RF amplitude of the octopole was set to 350 Vpp and its RF frequency to 5 MHz. In the transfer optics section, the time of flight was set to 1.2 ms and the frequency applied to the ICR transfer hexapole rods was set to 4 MHz. The excitation mode was set to sweep mode with a sweep step time of 15 µs. The ramped power excitation was chosen to be continuous (14–28%). The voltages of the ICR Paracell were chosen as follows: Transfer exit lens was set to −20 V and analyzer entrance voltage to −10 V. Front and Back Plate voltages were both set to 1.5 V. The side-Kick Offset was set to −1.5 V. Under these conditions a typical resolving power of 800,000 at m/z 400 was achieved. Prior to the measurement, external quadratic calibration was performed using the peptide calibration standard II (Bruker Daltonics) spiked with the small molecular mass compounds Olanzapine, Sunitinib, Romidepsin and Sorafenib. Additionally, mass spectra were acquired using a prominent signal at *m/z* 760.5851 ((PC(34:1) + H^+^) as a reference mass for single point online calibration. Mass tolerance for online calibration was set to 6 ppm. FTMS data were visualized using Bruker Compass DataAnalysis 4.4.

Solarix 7T XR FTICR was also used for fragmentation of candidate response lipid markers. The MALDI MS acquisition mode was set to tune to continuously acquire mass spectra. Quadrupole isolation was checked in the Source MS/MS tab. The isolation window was set to 1 *m/z*. Collision cell RF amplitude was adjusted to 2,000 V. RF frequency was set to 5. Next collision induced dissociation (CID) was selected. Collision energy was increased until the parent ion peak was reduced to 50% of its initial intensity. The remaining parameters were kept as described above.

### Processing of mass spectra and data analysis

Mass spectra were recalibrated and converted to text files in a flexAnalysis 3.4 (Bruker Daltonics) batch process. Mass spectra in text-file format were imported into R 3.3.1^31^ (R Foundation for Statistical Computing, Vienna, Austria) and processed using *MALDIquant* and, its companion, *MALDIquantForeign* packages^32^. The workflow consists of TIC-normalization, Top-Hat baseline subtraction, peak picking (method = “SuperSmoother”, halfWindowSize = 20, SNR >5), peak binning (tolerance = 0.002) and data reduction into a single feature matrix^32^. For multivariate data analysis, features were square root transformed. Principal component analysis (PCA) was calculated based on singular value decomposition after centering the variables towards zero. The *rgl* package was used for 3D-plotting of the PCA.

To identify drug-sensitive m/z features, a two-step feature filtration strategy was applied. First, the features were filtered by their variances across the whole dataset. Secondly the remaining features were filtered by their fit to a concentration-response curve.

For the first filtration step, feature-wise (i.e. *m/z*-wise) variances spanning all intensities depicted in the feature matrix were computed resulting in a one intensity-variance per *m/z* feature. Since the majority of the variances recorded were close to zero (i.e. *m/z* features unaffected by the drug), the variances produced a right-skewed variance-histogram (Suppl. Fig. S11). The mean of all intensity-variances, which in such situation falls to the right of the histogram peak, was selected as a threshold, above which feature-variances were considered to have significant changes occurring through all spectra.

In the second filtration step, the remaining features were fitted to a concentration-response curve. Signal intensities in the feature matrix of technical replicates were averaged. All descending curves were fitted to the equation:

1 $$f(x)=top+\frac{bottom-top}{1+{10}^{(LogIC50-x)\cdot HillSlope}}$$

using the nonlinear least squares regression implemented in R base package, where *f(x)* is the signal intensity, *x* is the decadal logarithm of the drug concentration, *top* is the *f(x)* value at the top plateau and *bottom* is the *f(x)* value at the bottom plateau. To calculate fits for ascending concentration-response curves, signals above a putative “toxic drug concentration”, i.e. signals corresponding to higher drug concentrations than those used for the maximum of a biphasic response, were excluded. Therefore, all concentrations after the highest data point were excluded. Moreover, a curve had to be defined by at least five data points, to exclude statistical noise. The remaining data points were fitted to the equation:

2 $$f(x)=bottom+\frac{top-bottom}{1+{10}^{(LogIC50-x)\cdot HillSlope}}$$

using the same nonlinear least squares regression method. Obtained peak lists were sorted by descending fit quality determined by coefficient of determination R^2^ value, excluding peaks with R^2^ values below 0.9. An isolated *m/z* value was considered as a response marker if its concentration response was reproduced in three biological replicate measurements.

### Data availability statement

Datasets generated during and/or analyzed during the current study are not publicly available but are available from the corresponding author on reasonable request.

## Electronic supplementary material

Supplementary Information
