# Supplementary material for: Automated analysis of lipid drug-response markers by combined fast and high-resolution whole cell MALDI mass spectrometry biotyping
Source: Sci Rep. 2018 Jul 26;8:11260. doi: 10.1038/s41598-018-29677-z (PMC6062520; doi:10.1038/s41598-018-29677-z)
Supplement: Supplementary file 1 — Supplementary Information [file 41598_2018_29677_MOESM1_ESM.pdf]

Automated analysis of lipid drug-response markers by combined fast and high-resolution whole cell MALDI mass spectrometry biotyping

David Weigt, Denis A. Sammour, Timon Ulrich, Bogdan Munteanu, Carsten Hopf

Table of contents

Supplementary Methods	Description of methods used for supplementary figures
Figure S1	Using DHB as matrix in the workflow leads to better separation in PCA space, i.e. a lower $J_{overlap}$ score, compared to ATT matrix.
Figure S2	No preference between direct sample resuspension in acetonitrile/H_20 versus additional hexane extraction.
Figure S3	Better separation of three cancer cell lines with “wet” matrix application protocols.
Figure S4	Addition of ammonium citrate results in comparable separation efficiency compared to no addition.
Figure S5	5,000 cells per measuring spot are required for effective suppression of matrix ions.
Figure S6	Reproducibility of WC-MALDI MS lipid/metabolite fingerprints.
Figure S7	Prolonged treatment with drug increases differences of lipid MS fingerprints of DMSO- and imatinib-treated cells.
Figure S8	Measurement of candidate response markers using ultra-high resolution FT-ICR MS.
Figure S9	No interfering signals for the low resolution features of HemeB and PC(36:1)+K+ observed in high resolution measurements.
Figure S10	Tyrosine kinase inhibitor, Dasatinib, concentration response.
Figure S11	*m/z* feature-wise variance analysis.
Table S1	Comparison of predicted and measured isotopic distribution of candidate drug response markers assessed by FT-ICR MS
Table S2	The potassium adduct of PC(36:1) enables monitoring of drug concentration-responses in K562 cells
Supplementary References	List of sources employed for the supplementary material

Supplementary Methods

HL60 and GIST T1 Cell culture

HL60 cells (ATCC, Rockville, USA) were cultivated in Iscove's Modified Dulbecco's Medium (IMDM) medium supplemented with 20 % fetal calf serum, 4.5 g L^{-1} glucose and 4 mM L-glutamine. The gastrointestinal stroma cell line GIST T1 was provided by the EU-MITIGATE consortium (www.mitigate-project.eu) and cultivated in IMDM supplemented with 1 mM L-glutamine, 15 % fetal calf serum (v v^{-1}) and 1x penicillin/streptomycin

Sample preparation using ATT-matrix

ATT-matrix was resuspended at 10 mg mL^{-1} in in 50% (v v^{-1}) acetonitrile in ddH2O supplemented with 0.5% TFA. Dried sample spots were spray-coated with DHB-matrix using a SunCollect sprayer. The spray protocol included a spray-head velocity set to 900 mm min^{-1} at a height of 2.8 cm. The distance between sprayed lines was 2 mm. The matrix flow rate was set to 30 µL min^{-1}.

Autoflex MALDI-TOF data acquisition

During method development the target was measured by an Autoflex speed MALDI-TOF mass spectrometer equipped with a 2 kHz Smartbeam II laser (Bruker Daltonics). Measurements were performed in reflector positive ion mode in a range of m/z 200-2,000. The sum of 4,000 laser shots per measuring spot was acquired at 40 different positions in random walk mode. The laser focus was set to "large". The sampling rate was 4 giga samples s^{-1}. Data acquisition was controlled by the AutoXecute function of the flexControl 3.4 software (Bruker Daltonics).

$J_{overlap}$ calculation

Within-class scatter (WS) and between-class scatter (BS) were calculated based on their implementation in the *DiscriMiner* package. The interclass overlap, $J_{overlap}$, a score that measures experimental repeatability and permits quantitative comparisons between different PCA coordinate systems, was calculated as described previously[1]:

$$J_{overlap} = \frac{|W_S|}{|B_S|}$$

The *rgl* package was used for 3D-plotting of the PCA, as well as drawing the distance lines between groups and ellipsoids around each group.

MSE_{mod} calculation

To evaluate matrix suppression and therefore a suitable on-target concentration of the respective sample, we used the matrix suppression effect for lipids (MSE_{lip})-score described earlier [2, 3] – with slight modifications: All peaks occurring in the matrix spectrum were matched with peaks of the sample spectra. The ratio of the sum of all peaks that were not matched with matrix peaks (∑no matrix) divided by the total amount of peaks (∑all peaks) was chosen as a modified MSE (MSE_{mod}):

$$MSE_{mod} = \frac{\sum no\ matrix}{\sum all\ peaks}$$

Supplementary Data

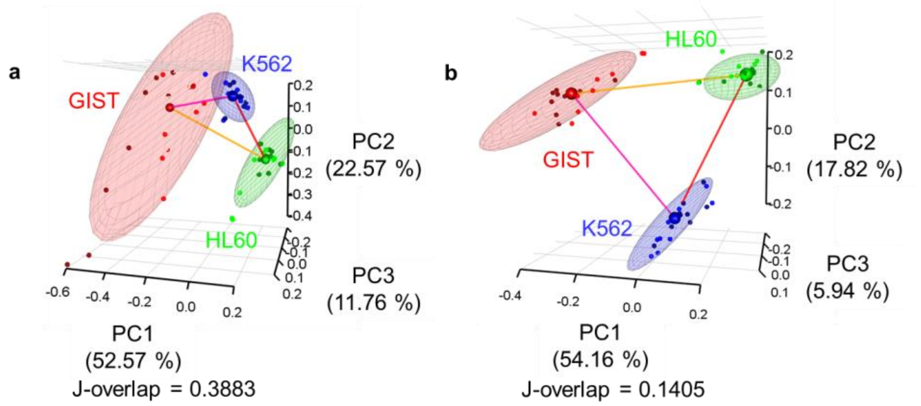

Figure S1. Using DHB as matrix in the workflow leads to better separation in PCA space, i.e. a lower $J_{overlap}$ score, compared to ATT matrix. Cell pellets of three different cell lines (GIST T1, HL60 and K562) were resuspended at 5,000 cells µL^{-1} in acetonitrile/ddH$_2$0. One microliter of the suspension was applied to a MALDI target plate, which was spray-coated with either ATT (**a;** 10 mg mL^{-1} in acetonitrile/ddH$_2$0/TFA (60/40/0.5)) or DHB (**b;** 20 mg mL^{-1} in the same solvent). Samples were measured using an Autoflex Speed MS. PCAs and variances were calculated in R. Biological replicates are colored in different shades of the same color (mass range: 200-2000, S/N > 5, N = 2 biological replicates, each with ≥ 8 technical replicates measured on the same day).

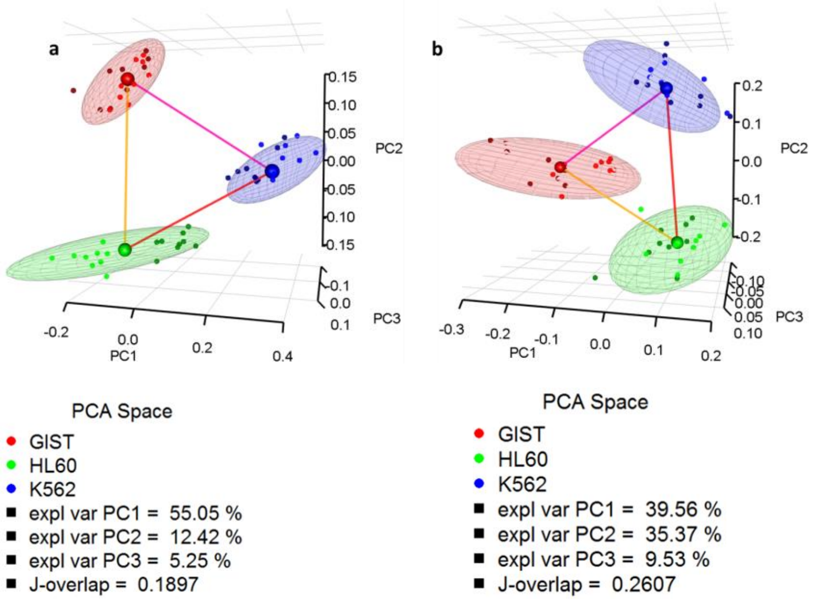

Figure S2. No preference between direct sample resuspension in acetonitrile/H$_2$0 versus additional hexane extraction. (**a**) Cell pellets of three different cell lines (GIST, HL60 and K562) were resuspended at 5,000 cells µL^{-1} in acetonitrile/H$_2$0 (1 to 1). 1 µL of the suspension was applied on the target plate (5,000 cells spot^{-1}). (**b**) Hexane was added 1to 1 to the acetonitrile/ddH$_2$0 suspension. Samples were vortexed for 30 seconds. For phase separation, samples were centrifuged for 30 seconds at 14,000 rpm. 1 µL of the hexane extract was applied on the MTP. Cells were spray-coated with DHB matrix: 20 mg mL^{-1} in acetonitrile/ddH$_2$0/TFA (60/40/0.5). Samples were measured using an Autoflex Speed MALDI-TOF MS. PCA and variances were calculated in R. Biological replicates were colored in different shade of the same color (*m/z* 200-2000, S/N > 5, n = 2 biological replicates with respectively eight technical replicates measured on the same day).

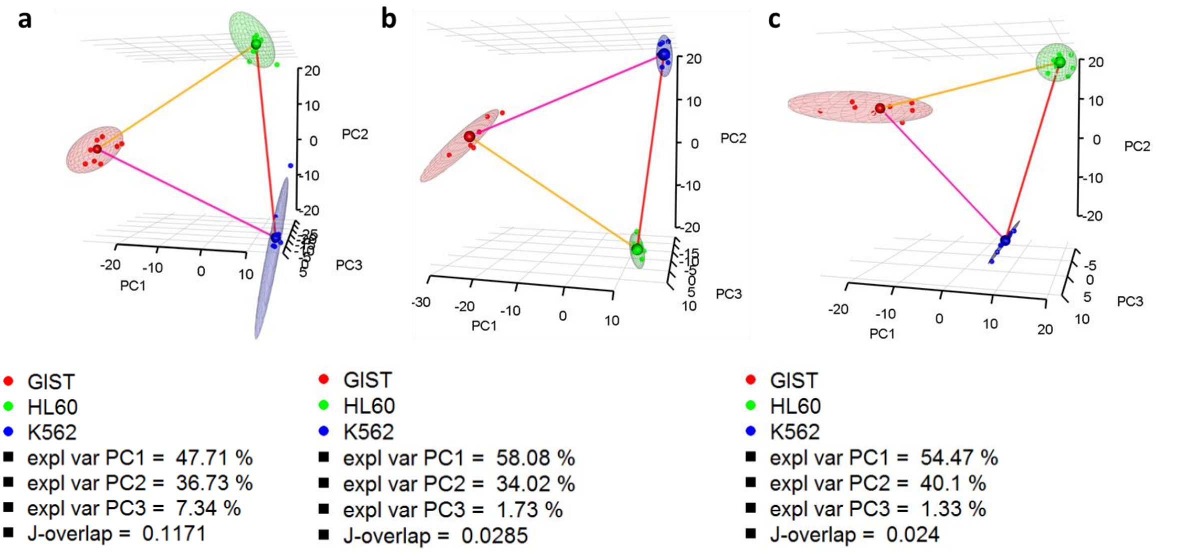

Figure S3. Better separation of three cancer cell lines with "wet" matrix application protocols. Cell pellets of three different cell lines (GIST, HL60 and K562) were resuspended at 5,000 cells µL^{-1} in acetonitrile/dd$H_2$0 (1 to 1). 1 µL of the suspension was applied on the target plate (5,000 cells/spot). Matrix (20 mg mL^{-1} DHB in 50% acetonitrile, 0.2% TFA) was applied following different spray-protocols with the SunCollect sprayer. Motor axis of spray head was either moved fast (1250 mm min^{-1}) or slow (900 mm min^{-1}). Matrix flow rate was either adjusted to "dry" (15 µL min^{-1}) or to "wet" (30 µL min^{-1}). Samples were measured using an ultraflextreme MALDI-TOF MS. The illustrated PCAs of samples prepared following a fast-dry (**a**), a fast-wet (**b**), and a slow-wet (**c**) spray protocol were calculated in R (*m/z* 200-2000, S/N > 5, n ≥ 8 technical replicates measured on the same day). J-overlap values suggest that "wet" matrix application protocols are preferred.

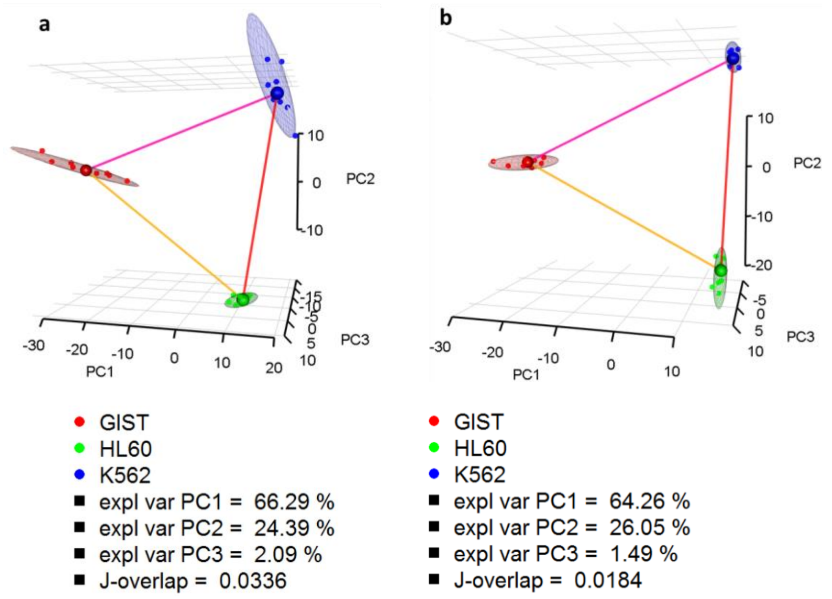

Figure S4. Addition of ammonium citrate results in comparable separation efficiency compared to no addition. Cell pellets of three different cell lines (GIST, HL60 and K562) were resuspended at 5,000 cells µL^{-1} in either (**a**) acetonitrile/$H_2$0 (1/1) or (**b**) acetonitrile/30 mM ammonium citrate (1/1). 1 µL of the suspension was applied on the target plate (5,000 cells/spot). Cells were spray-coated with DHB matrix (20 mg mL^{-1} in acetonitrile/$H_2$0/TFA (60/40/0.5)). Samples were measured using an ultraflextreme MS. PCA and variances were calculated in R (n ≥ 8 technical replicates measured on the same day).

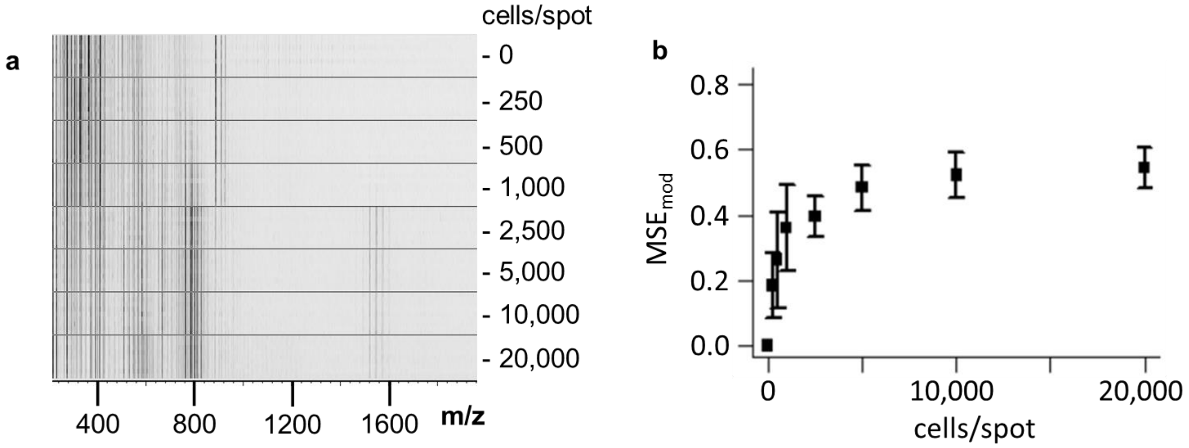

Figure S5. 5,000 cells per measuring spot are required for effective suppression of matrix ions. K562 cells were diluted in acetonitrile/ddH$_2$0 (1 to 1). One microliter of the cell suspension was applied to a MALDI target plate, which was then spray-coated with 20 mg mL^{-1} DHB dissolved in acetonitrile / ddH$_2$O / TFA. 4,000 shots per spot were acquired in a random walk in reflector positive ion mode. (**a**) Spectra of a K562 cell dilution series are illustrated as gel view generated by ClinProTools software. (N = 8 technical replicates per group measured on the same day). (**b**) The modified matrix suppression effect (MSE_{mod}) score was plotted against the cell number per measuring spot (mean ± standard deviation for N=3 technical replicates prepared on different days).

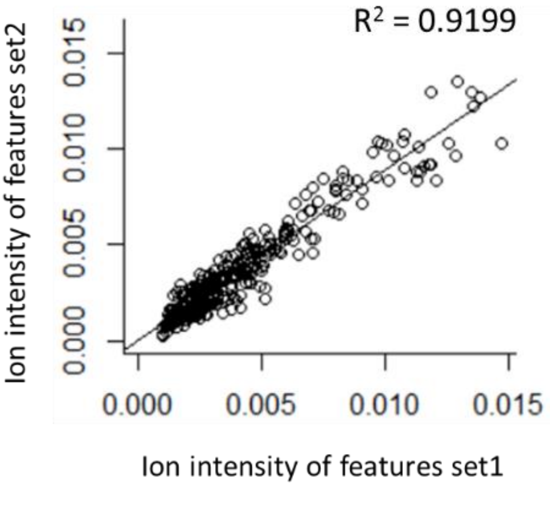

Figure S6. Reproducibility of WC-MALDI MS lipid/metabolite fingerprints. Mean intensities of all peaks (S/N > 10, *m/z* 500-2000) for the acetonitrile/ddH$_2$0 (1 to 1) preparation were plotted for two replicates measured on two different days. Peaks with intensities above the 95% percentile (0.0166) were excluded. The coefficient of determination (R^2) was 0.92.

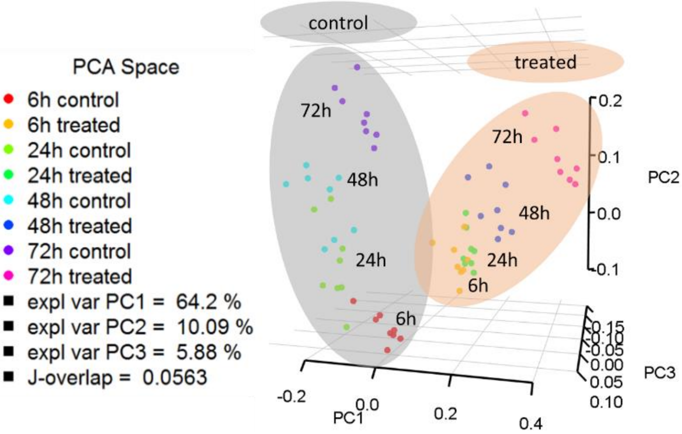

Figure S7. Prolonged treatment with drug increases differences of lipid MS fingerprints of DMSO- and imatinib-treated cells. K562 cells were treated with imatinib in a time course experiment. Imatinib treatment was stopped by cell harvest after 6, 24, 48 and 72 h. Samples were measured by an ultrafleXtreme MALDI-TOF MS in reflector positive ion mode. Feature extraction and PCA calculation was performed in R. After 48 h a clear separation of treated and untreated cells in PCA space was observed (N=8 technical replicates measured on the same day).

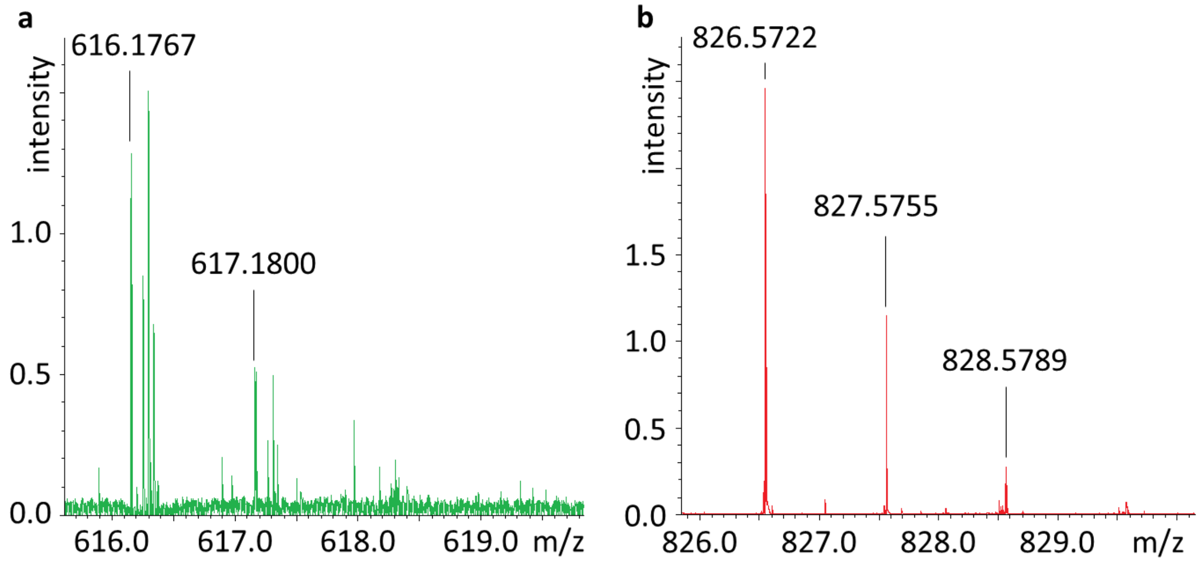

Figure S8. Measurement of candidate response markers using ultra-high resolution FT-ICR MS. Spots that show highest feature of interest intensity were re-measured using a SolariX 7T XR FTICR mass spectrometer. For this reason a spot containing cells treated with 1 µM Imatinib was used for re-measurement of the MALDI-TOF MS feature *m/z* 616.2 (**a**) and a spot containing DMSO-treated cells was used for re-measurement of the MALDI-TOF MS feature *m/z* 826.6 (**b**).

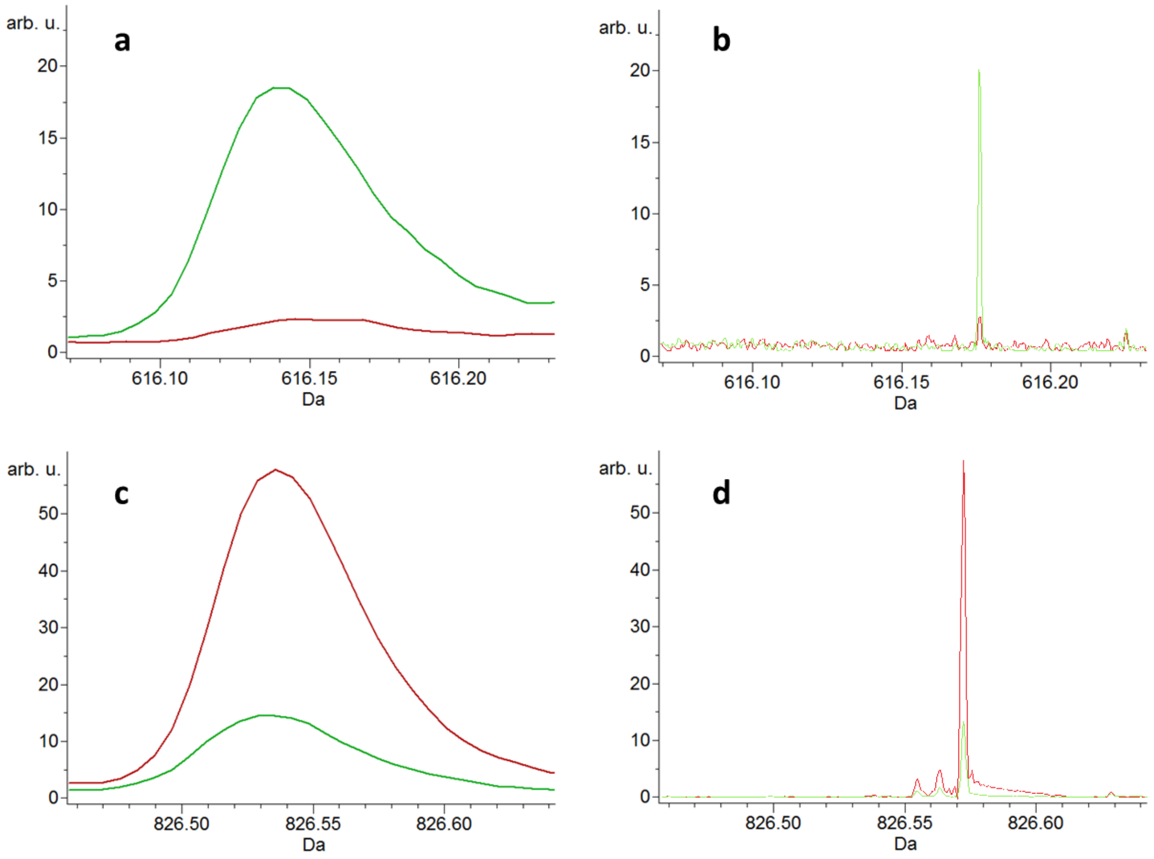

Figure S9. No interfering signals for the low resolution features of Heme B and PC(36:1)+K$^+$ observed in high resolution measurements. Mass spectra of cells treated with 1 µm imatinib are colored in green and DMSO-treated in red. Displayed are zoom ins of heme B (**a**) and PC(36:1)+K$^+$ (**c**) measured using low resolution MALDI-TOF and the respective mass ranges of high resolution MALDI-FTICR remeasurements (**b**,**d**). TOF-spectra display the average of 8 replicate measurements. FTICR-spectra are single measurements.

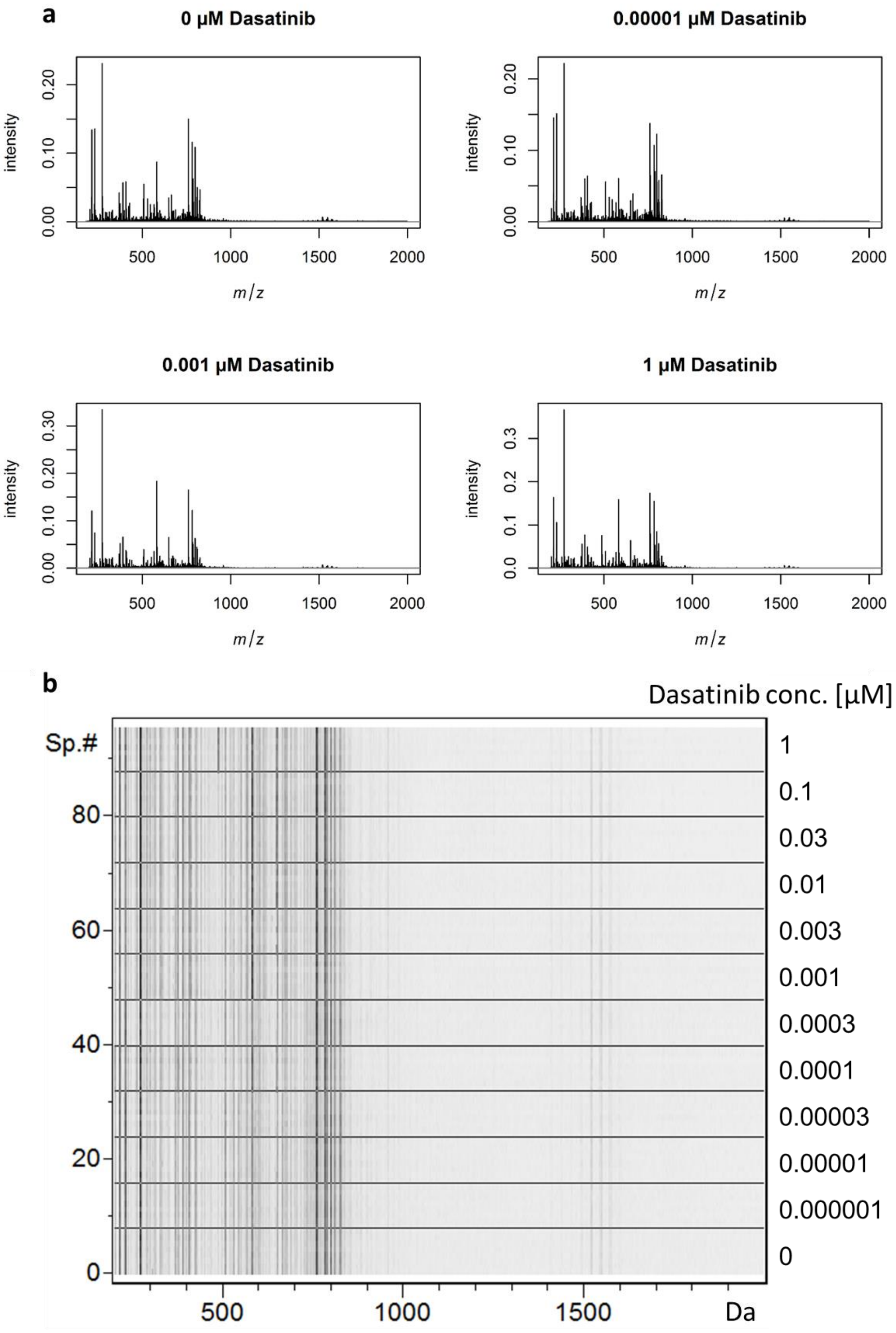

Figure S10. Tyrosine kinase inhibitor, Dasatinib, concentration response. Mass spectra of cells treated with the indicated concentrations of Dasatinib illustrated as both single spectra (**a**, average of 8 technical replicate measurements) and gel view (**b**, each stack represents a single replicate measurement).

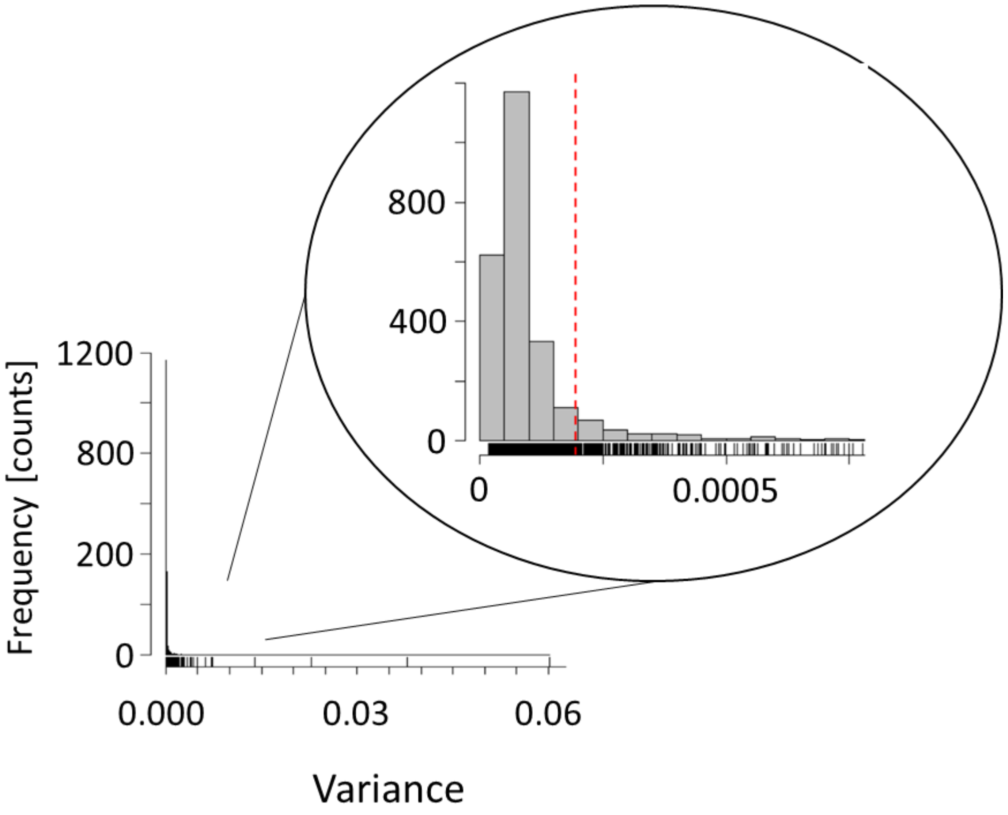

Figure S11. *m/z* feature-wise variance analysis. K562 cells were seeded at 0.25*10^6 cells mL^{-1} in 24-well plates. The following day the cells were treated with various concentrations of Imatinib. Cell pellets were resuspended in 100 µL acetonitrile / water (1 to 1). Eight replicates of 1 µL of the solution were applied to a 384-spot ground steel target plate. Dried sample spots were spray-coated with DHB-matrix and measured by an ultrafleXtreme in reflector positive ion mode. Spectra were calibrated and baseline-subtracted using flexAnalysis software. R computing language was used for feature extraction. The histogram illustrates the intensity-variances of *m/z* features among all groups treated with different concentrations of imatinib. The dashed red line represents the mean of all variances which, in such a right-skewed histogram, falls to the right of the majority low-variance bins. The *m/z* features for which the variance was higher than this threshold were considered significant.

a

isotopes<br>A	pred. rel. abundance	meas. rel. abundance
**616.1767**	100	100
**617.1801**	36.8	39.1

b

isotopes	pred. rel. abundance	meas. rel. abundance
**826.5723**	100	100
**827.5756**	47.9	47.3
**828.5790**	11.2	11.5

Table S1: Comparison of predicted and measured isotopic distribution of candidate drug response markers assessed by FT-ICR MS. (**a**) The isotopic distribution of heme B ($C_{34}H_{32}O_4N_4Fe$) was simulated for a hypothetic resolution of 545,138 (pred. rel. abundance), which matches the resolution of the signal at *m/z* 616.1767 in the mass spectrum. The predicted relative abundance was compared to the measured relative (meas. rel.) abundance. (**b**) Accordingly isotopic distribution of PC(36:1) + K^+ ($C_{44}H_{86}NO_8PK$) was simulated for a resolution of 409,658, which matches the resolution of the signal at *m/z* 826.5722.

**Inhibitor**	***m/z* – 826.6**<br>**pIC_{50}**
**Imatinib**	6.6 ± 0.1
**Dasatinib**	9.6 ± 0.1
**Nilotinib**	8.3 ± 0.1
**Vandetanib**	5.7 ± 0.2
**Sunitinib**	5.2 ± 0.1
**Chloroquine**	-

Table S2. The potassium adduct of PC (36:1) enables monitoring of drug concentration-responses in K562 cells. Table of pIC_{50} values for each inhibitor derived from measures of the decrease of *m/z* 826.6 upon treatment with BCR-Abl inhibitors and controls (mean ± standard deviation for N=3 biological replicates prepared on different days).

Supplementary References

[1] K. Erich, D.A. Sammour, A. Marx, C. Hopf, Scores for standardization of on-tissue digestion of formalin-fixed paraffin-embedded tissue in MALDI-MS imaging, Biochim Biophys Acta, 1865 (2017) 907-915.

[2] G. McCombie, R. Knochenmuss, Small-molecule MALDI using the matrix suppression effect to reduce or eliminate matrix background interferences, Anal Chem, 76 (2004) 4990-4997.

[3] A. Fulop, M.B. Porada, C. Marsching, H. Blott, B. Meyer, S. Tambe, R. Sandhoff, H.D. Junker, C. Hopf, 4-Phenyl-alpha-cyanocinnamic acid amide: screening for a negative ion matrix for MALDI-MS imaging of multiple lipid classes, Anal Chem, 85 (2013) 9156-9163.
